# Outcomes following different thermal ablation strategies in patients with oligometastatic colorectal lung metastases

**DOI:** 10.3389/fonc.2026.1902067

**Published:** 2026-07-08

**Authors:** Youzhuo Quan, Tao Xu, Yunan Xiang, Kun Liu

**Affiliations:** 1 Department of Cardiology, Union Hospital, Tongji Medical College, Huazhong University of Science and Technology, Wuhan, China; 2 Hubei Key Laboratory of Biological Targeted Therapy, Union Hospital, Tongji Medical College, Huazhong University of Science and Technology, Wuhan, China; 3 Hubei Engineering Research Center for Immunological Diagnosis and Therapy of Cardiovascular Diseases, Union Hospital, Tongji Medical College, Huazhong University of Science and Technology, Wuhan, China; 4 Department of Radiology, Union Hospital, Tongji Medical College, Huazhong University of Science and Technology, Wuhan, China; 5 Hubei Provincial Clinical Research Center for Precision Radiology & Interventional Medicine, Wuhan, China; 6 Hubei Provincial Key Laboratory of Molecular Imaging, Wuhan, China; 7 Department of Radiology, The Second Affiliated Hospital and Yuying Children's Hospital of Wenzhou Medical University, Wenzhou, Zhejiang, China

**Keywords:** colorectal cancer, lung metastases, systemic therapy, thermal ablation, treatment sequencing

## Abstract

**Purpose:**

Image-guided thermal ablation (IGTA) is increasingly integrated with systemic therapy for oligometastatic colorectal cancer with lung metastases, but the optimal timing of ablation relative to systemic treatment remains unclear. This study evaluated whether treatment sequencing affects survival and local tumor control.

**Methods:**

This multicenter retrospective cohort study included 289 patients with oligometastatic colorectal lung metastases who underwent CT-guided percutaneous thermal ablation between April 2015 and April 2023. Patients were classified into delayed-ablation, simultaneous-ablation, and instant-ablation groups according to the timing of ablation relative to systemic therapy. Overall survival (OS), progression-free survival (PFS), and local tumor progression-free survival (LTPFS) were assessed using Cox regression and inverse probability of treatment weighting (IPTW) based on the average treatment effect framework.

**Results:**

The delayed-, simultaneous-, and instant-ablation groups included 118, 87, and 84 patients, respectively. The primary technical success rate was 97.5%, and secondary technical success reached 100%. After IPTW adjustment, simultaneous ablation was associated with a lower risk of death than delayed ablation (HR, 0.51; 95% CI, 0.27-0.96; P = 0.037), whereas instant ablation showed no OS advantage. Simultaneous ablation showed a trend toward improved PFS (HR, 0.75; 95% CI, 0.55-1.03; P = 0.076). instant ablation was associated with a higher risk of local tumor progression (HR, 2.62; 95% CI, 1.28-5.37; P = 0.008). No treatment-related deaths occurred.

**Conclusion:**

Simultaneous ablation was associated with longer OS in patients with oligometastatic colorectal lung metastases, whereas instant ablation may be associated with poorer local control. Prospective validation is warranted.

## Introduction

The lung is one of the most common sites of distant metastasis in colorectal cancer. With advances in systemic therapy, molecular profiling, and local treatment techniques, a subset of patients with metastatic colorectal cancer are no longer treated solely with palliative intent but are considered for multimodal strategies aimed at long-term survival or durable no evidence of disease (NED) (1, 2). Surgical resection and local ablation may prolong survival in carefully selected patients with oligometastatic disease (3). Guidelines, including those from ESMO, recommend that patients with controlled primary tumors and limited pulmonary oligometastases be considered for local therapy, including surgery, thermal ablation, or stereotactic radiotherapy, within a multidisciplinary framework and in conjunction with systemic treatment (4–6).

Among available local treatment options, pulmonary metastasectomy remains the conventional curative-intent approach, although not all patients are suitable surgical candidates. Image-guided thermal ablation (IGTA) has therefore emerged as an important local treatment for colorectal lung metastases because it is minimally invasive, repeatable, relatively lung-sparing, and readily integrated with other therapies (7). Recent studies suggest that, in carefully selected patients, thermal ablation can achieve long-term oncologic outcomes approaching those of surgery (8, 9); furthermore, adding local ablative therapy to standard systemic therapy may further improve long-term survival (10). A meta-analysis likewise reported superior long-term outcomes with multimodal treatment compared with single-modality strategies (11). Collectively, these data have moved IGTA earlier in the multidisciplinary treatment pathway.

Current clinical discussions have focused largely on whether local therapy should be added; however, for patients with colorectal lung metastases undergoing IGTA, direct comparative evidence remains limited regarding which sequence of ablation and systemic therapy is associated with better outcomes.

We therefore conducted a multicenter retrospective cohort study to evaluate how the timing of first pulmonary ablation relative to systemic therapy affects survival and recurrence in patients with oligometastatic colorectal lung metastases. By integrating local ablation, systemic therapy, and molecular stratification, this study sought to define a clinically actionable framework for treatment sequencing, refine risk stratification, and support individualized treatment planning.

## Materials and methods

### Study design and patients

This multicenter retrospective cohort study included patients who underwent CT-guided percutaneous thermal ablation for pulmonary metastases at three regional medical centers between April 2015 and April 2023. The study was approved by the ethics committees of the participating centers (approval No. IEC-2024-1074), and the requirement for informed consent was waived because of the retrospective design; the study was conducted in accordance with the Declaration of Helsinki.

The analysis focused on patients with oligometastatic colorectal cancer involving the lung. Oligometastatic disease was defined as no more than five pulmonary metastases, a maximum pulmonary lesion diameter of <50 mm, and the feasibility of definitive local treatment or durable disease control for all known metastatic sites as determined by MDT review.

Limited extrapulmonary metastases (mainly liver metastases) were allowed only when they were technically treatable by surgery, ablation, radiotherapy, or other local therapy, or when they remained radiologically controlled under systemic therapy. Patients with diffuse pulmonary metastases, extensive peritoneal disease, uncontrolled multiple-organ metastases, or rapidly progressive extrapulmonary disease were excluded.

The inclusion criteria were as follows: pathologically confirmed primary colorectal cancer; pulmonary metastases confirmed by pathology or typical imaging follow-up; no previous CT-guided thermal ablation intended for local cure or tumor burden clearance; suitability for local treatment as assessed by the multidisciplinary team (MDT); previous R0 resection of the primary tumor; and an Eastern Cooperative Oncology Group (ECOG) performance status of 0-2.

**Figure 1:**
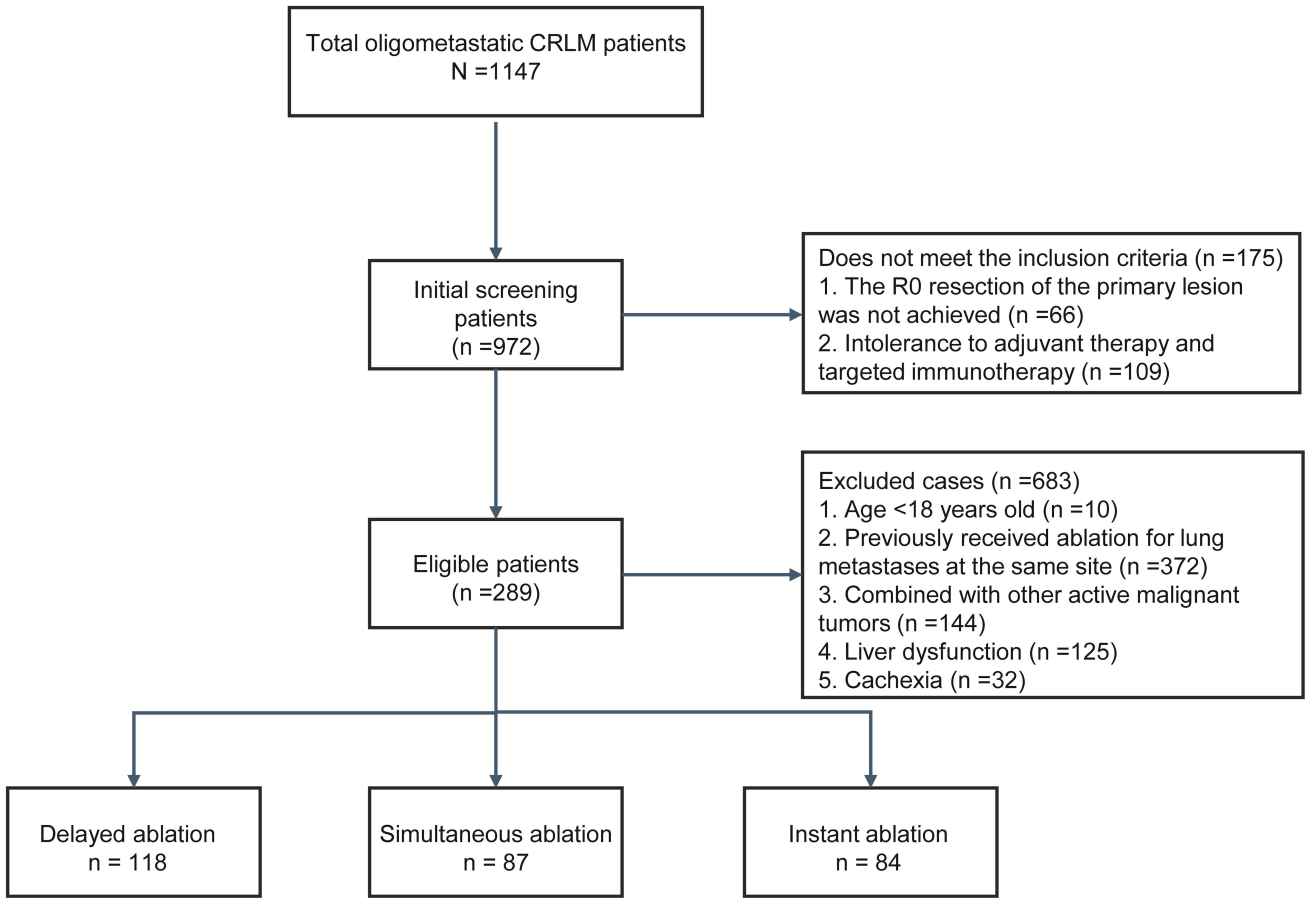
Flowchart for inclusion and exclusion criteria.

The exclusion criteria were diffuse pulmonary metastases or uncontrolled extensive extrapulmonary disease; prior ablation of a pulmonary metastasis at the same site; age younger than 18 years; another active malignancy; severe cardiopulmonary dysfunction precluding ablation or systemic therapy; and Child-Pugh class C liver function (**Figure 1**).

### Ablation procedure and periprocedural management

Treatment plans were determined after multidisciplinary evaluation involving interventional radiology, medical oncology, anesthesiology, and thoracic surgery. Preprocedural evaluation included pulmonary function testing, electrocardiography, anesthetic assessment, and biochemical testing, and all patients provided written informed consent before treatment.

All ablation procedures were performed by interventional radiologists with more than 6 years of experience. Patient positioning was selected according to lesion location, and procedures were performed under local anesthesia with conscious sedation and analgesia. CT was used to plan the puncture route, and biplanar or multiphase scans were obtained to confirm the needle trajectory and avoid critical structures, including major vessels, bronchi, and pleura. During the procedure, intermittent or real-time CT was used to monitor probe position and the ablation zone, and energy delivery was adjusted according to impedance feedback. Vital signs were monitored continuously throughout the procedure.

Primary technical success was defined as complete coverage of the target lesion by a single ablation session on the initial DCE-CT, with an ablation margin of at least 5 mm, consistent with previous studies or guidelines (12, 13). CT was repeated within 24 hours after the procedure to assess technical success and early complications. Secondary technical success was defined as complete ablation after additional treatment of residual disease.

### Imaging assessment and ablation margin assessment

All ablation procedures and follow-up assessments were reviewed using CT images obtained as part of routine clinical care. Technical efficacy was evaluated on contrast-enhanced CT performed within 24 hours after ablation. The CT protocol included thin-section acquisition with multiplanar reconstruction; slice thickness was 1.5 mm for ablation planning and 1.5 mm for post-ablation assessment. Contrast-enhanced images were obtained unless contraindicated.

Ablation margins were retrospectively assessed on the first post-ablation contrast-enhanced CT used for technical efficacy evaluation. Pre-ablation CT images were reviewed side by side with post-ablation images on PACS. The minimal ablation margin was defined as the shortest distance from the boundary of the original tumor to the outer edge of the ablation zone. Measurements were performed on two-dimensional axial CT images. Coronal and sagittal reconstructions were used for anatomic reference when available.

Ablation margins were retrospectively measured by one experienced radiologist and then reviewed by a second radiologist. Margin adequacy was categorized as adequate (≥5 mm), insufficient (<5 mm), or not assessable. Discrepant measurements were resolved by consensus. Technical success was assessed on the 24-hour post-ablation CT images with review by an experienced radiologist/interventional radiologist other than, or in addition to, the treating operator; uncertain cases were resolved by consensus.

### Somatic mutation analysis

After pathological review, formalin-fixed, paraffin-embedded (FFPE) tumor tissue blocks were selected for molecular testing. Genomic DNA was extracted using the QIAamp DNA FFPE Tissue Kit (Qiagen, Hilden, Germany). Next-generation sequencing (NGS) was then performed on the Illumina MiSeq platform (Illumina, Hayward, CA, USA) (14). MSI status was determined using a previously reported and validated analytical pipeline based on the distribution of sequencing read counts.

### Definition of treatment timing

The treatment plan was determined at a local multidisciplinary meeting involving surgeons, oncologists, gastroenterologists, radiologists, and radiotherapists, and was finalized in accordance with the patient’s preferences. Systemic treatment cycles were defined according to the standard dosing schedule of the first-line regimen actually received, including FOLFOX, FOLFIRI, CAPOX, and FOLFOXIRI, with or without bevacizumab or cetuximab.

We used timing-based group names to distinguish whether ablation was performed after induction systemic therapy, during an ongoing systemic treatment course, or before initiation of systemic therapy. Patients were classified into three groups according to the temporal relationship between ablation and systemic therapy (15).

Delayed-ablation group: patients received 2 to 4 weeks’ systemic therapy first and then underwent ablation of pulmonary metastases after imaging assessment.

Simultaneous -ablation group: ablation was performed during an ongoing systemic treatment course, within the prespecified interval from the most recent systemic therapy cycle, and systemic therapy was continued after ablation.

Instant-ablation group: patients first underwent ablation of lung metastases and then started systemic treatment 2 to 4 weeks later.

The three groups of patients repeated the systemic treatment cycle every 2 or 3 weeks for approximately 6 months (the delayed ablation group continued systemic treatment after ablation).

### Follow-up

The primary endpoints were overall survival (OS), progression-free survival (PFS), and local tumor progression-free survival (LTPFS). OS was defined as the time from first ablation of pulmonary metastases to death from any cause. PFS was defined as the time from first ablation to radiologic progression at any site or death. LTPFS was defined as the time from first ablation to local tumor progression within or along the margin of the ablation zone.

Patients underwent DCE-CT 3–4 weeks after ablation to assess technical efficacy, followed by contrast-enhanced chest and abdominal CT every 3–6 months during the first 2 years and every 6–12 months thereafter. Patients with local or systemic progression received repeat ablation, surgery, radiotherapy, or subsequent systemic therapy according to their clinical condition.

### Statistical analysis

Because the proportion of missing data was less than 20% for all variables, missing values were handled using multiple imputation by chained equations (MICE), with 10 imputations; subsequent analyses were performed on the pooled imputed data (**Supplementary Table 1**). Continuous variables were summarized as means +/- standard deviations or medians with interquartile ranges (IQRs), as appropriate, and categorical variables were summarized as counts and percentages. Between-group comparisons were performed using analysis of variance, the Kruskal-Wallis test, the chi-square test, or Fisher’s exact test, as appropriate. Cox proportional hazards models were constructed to identify clinical factors associated with OS, PFS, and LTPFS. Variables with P < 0.20 in univariate analyses were entered into the multivariable models. To compare the independent associations of the three timing strategies with outcomes, inverse probability of treatment weighting (IPTW) based on the average treatment effect (ATE) was further applied. Propensity scores for the three treatment groups were estimated using multinomial logistic regression, incorporating sex, age, primary tumor site, differentiation state, T designation, extrapulmonary metastasis, CEA >= 20 ng/mL, CA19-9 >= 40 U/mL, number of pulmonary metastases, maximum lesion diameter, close to perivascular, close to peribronchial, hilar and mediastinal lymph node metastases, RAS/BRAF status, and microsatellite status to generate individual weights. Covariate balance was assessed using absolute standardized mean differences (SMDs), with an absolute SMD < 0.10 indicating adequate balance. Outcomes were compared using weighted Cox proportional hazards models, and standard errors were estimated by bootstrap resampling (R = 500). Weighted Kaplan-Meier curves were generated using the IPTW-KM method, and 95% confidence intervals were obtained by bootstrap resampling. LTPFS was treated as the event of interest, and death before LTPFS was treated as the competing event. Fine-Gray subdistribution hazard regression was used to estimate subdistribution hazard ratios (SHRs) and 95% confidence intervals, using delayed ablation as the reference group. To remain consistent with the primary weighted survival analyses, an IPTW-weighted Fine-Gray model was additionally fitted. Standard errors and confidence intervals were estimated using patient-level bootstrap resampling with 500 replicates. All tests were two-sided, and P < 0.05 was considered statistically significant. Statistical analyses were performed using R software, version 4.5.2.

## Results

### Patient characteristics and technical efficacy

A total of 289 patients were included, and their baseline demographic and clinical characteristics are summarized in **Table 1**. Of these, 118 patients were assigned to the delayed-ablation group, 87 to the simultaneous -ablation group, and 84 to the instant-ablation group. Overall, 166 patients (57.4%) were male and 123 (42.6%) were female. The median ages were 61 years (IQR, 13), 58 years (IQR, 10), and 61 years (IQR, 12), respectively, with no significant between-group differences in age or sex distribution (P = 0.788 and P = 0.571, respectively). Baseline oncologic characteristics, including primary tumor location (rectum vs colon), differentiation grade, T stage, and the proportion of synchronous metastases, were comparable across the three groups (all P > 0.05); overall, colon primary tumors accounted for 60.9% of cases (176/289), and T3-T4 disease accounted for 84.4% (244/289). In addition, the distributions of RAS/BRAF mutation status and microsatellite status were similar among the groups (all P > 0.05). Notably, baseline CA19–9 levels differed significantly among the groups [median (IQR), 19 (20) vs 15 (24) vs 14 (21); P = 0.032], and the proportion of lesions adjacent to vessels was highest in the instant-ablation group (11.9% vs 20.7% vs 26.2%; P = 0.031); no other baseline variables differed significantly. The median minimal ablation margins were 6.7 mm, 7.0 mm, and 7.5 mm in the delayed-, simultaneous -, and instant-ablation groups, respectively, with no significant between-group difference (P = 0.234). The distribution of adequate versus insufficient margins did not differ significantly among groups (P = 0.350). The first postprocedural DCE-CT assessment showed that most lesions were completely ablated after a single session, yielding a primary technical success rate of 97.5%. After additional IGTA was performed for the 13 lesions with residual disease after the initial session, complete ablation was achieved in all cases, corresponding to a secondary technical success rate of 100% (**Supplementary Table 2**).

**Table 1:** Baseline characteristics of patients in different treatment sequence groups.

Characteristic	Timing of ablation and systemic therapy			p-value
	Delayed ablation n = 118	Simultaneous ablation n = 87	Instant ablation n = 84	
Sex, n (%)				0.571^1^
Male	67 (56.8%)	47 (54.0%)	52 (61.9%)	
Female	51 (43.2%)	40 (46.0%)	32 (38.1%)	
Age, Median (IQR)	61 (13)	58 (10)	61 (12)	0.788^2^
Primary tumor site, n (%)				0.398^1^
Rectum	42 (35.6%)	39 (44.8%)	32 (38.1%)	
Colon	76 (64.4%)	48 (55.2%)	52 (61.9%)	
Differentiation state, n (%)				0.529^1^
Low	28 (23.7%)	23 (26.4%)	24 (28.6%)	
Medium	52 (44.1%)	45 (51.7%)	37 (44.0%)	
High	38 (32.2%)	19 (21.8%)	23 (27.4%)	
T designation, n (%)				0.856^1^
T1-T2	19 (16.1%)	12 (13.8%)	14 (16.7%)	
T3-T4	99 (83.9%)	75 (86.2%)	70 (83.3%)	
Synchronous metastases, n (%)	26 (22.0%)	20 (23.0%)	27 (32.1%)	0.224^1^
CEA, Median (IQR)	4 (8)	5 (8)	3 (4)	0.058^2^
CA199, Median (IQR)	19 (20)	15 (24)	14 (21)	0.032^2^
Number of extrapulmonary metastases, n (%)				0.687^3^
0	86 (72.9%)	60 (69.0%)	59 (70.2%)	
1	23 (19.5%)	22 (25.3%)	21 (25.0%)	
2	8 (6.8%)	4 (4.6%)	2 (2.4%)	
3	1 (0.8%)	1 (1.1%)	2 (2.4%)	
Ablation method, n (%)				0.076^1^
RFA	60 (50.8%)	32 (36.8%)	32 (38.1%)	
MWA	58 (49.2%)	55 (63.2%)	52 (61.9%)	
Maximum diameter of the lesion, Median (IQR)	15 (18)	12 (17)	12 (14)	0.319^2^
Number of pulmonary metastases, n (%)				0.522^1^
Single	57 (48.3%)	49 (56.3%)	44 (52.4%)	
Multiple	61 (51.7%)	38 (43.7%)	40 (47.6%)	
Close to perivascular, n (%)	14 (11.9%)	18 (20.7%)	22 (26.2%)	0.031^1^
Close to peribronchial, n (%)	17 (14.4%)	10 (11.5%)	16 (19.0%)	0.375^1^
Hilar lymph node metastasis, n (%)	19 (16.1%)	14 (16.1%)	8 (9.5%)	0.347^1^
Mediastinal lymph node metastasis, n (%)	22 (18.6%)	9 (10.3%)	7 (8.3%)	0.066^1^
Pneumothorax, n (%)	41 (34.7%)	35 (40.2%)	42 (50.0%)	0.093^1^
RAS mutation, n (%)	55 (46.6%)	36 (41.4%)	32 (38.1%)	0.466^1^
BRAF mutation, n (%)	13 (11.0%)	6 (6.9%)	7 (8.3%)	0.576^1^
Microsatellite stability, n (%)				0.774^3^
MSS	99 (83.9%)	75 (86.2%)	69 (82.1%)	
MSI-H	17 (14.4%)	9 (10.3%)	13 (15.5%)	
MSI-L	2 (1.7%)	3 (3.4%)	2 (2.4%)	
Adjuvant chemotherapy, n (%)				0.877^1^
FOLFOX	45 (38.1%)	31 (35.6%)	29 (34.5%)	
FOLFIRI	36 (30.5%)	30 (34.5%)	31 (36.9%)	
CAPOX	18 (15.3%)	9 (10.3%)	9 (10.7%)	
FOLFOXIRI	19 (16.1%)	17 (19.5%)	15 (17.9%)	
Targeted therapy, n (%)				0.294^1^
No application	18 (15.3%)	10 (11.5%)	6 (7.1%)	
Anti-VEGF drug	36 (30.5%)	35 (40.2%)	29 (34.5%)	
Anti-EGFR drug	64 (54.2%)	42 (48.3%)	49 (58.3%)	

CEA, Carcinoembryonic Antigen; CA19-9, Carbohydrate Antigen 19-9; RFA, Radiofrequency Ablation; MWA, Microwave Ablation; RAS, Rat Sarcoma viral oncogene homolog; BRAF, v-Raf murine sarcoma viral oncogene homolog B; MSS, Microsatellite Stable; MSI-H, Microsatellite Instability-High; MSI-L, Microsatellite Instability-Low; FOLFOX, Folinic acid (Leucovorin) + Fluorouracil (5-FU) + Oxaliplatin; FOLFIRI, Folinic acid (Leucovorin) + Fluorouracil (5-FU) + Irinotecan; CAPOX, Capecitabine + Oxaliplatin; FOLFOXIRI, Folinic acid + Fluorouracil + Oxaliplatin + Irinotecan; EGFR, Epidermal Growth Factor Receptor; VEGF, Vascular Endothelial Growth Factor.

^1^ Pearson’s Chi-squared test.

^2^ Kruskal-Wallis rank sum test.

^3^ Fisher’s exact test.

### Univariate and multivariable Cox regression

Univariate Cox regression analysis (**Supplementary Table 3**) showed that adverse factors for OS and PFS were mainly related to tumor burden and molecular features, including maximum lesion diameter, metastatic burden, and RAS/BRAF status, whereas targeted therapy and certain treatment-timing strategies were associated with lower event risks. For LTPFS, RAS mutation and instant ablation were associated with a higher risk of local progression, whereas anti-VEGF and anti-EGFR therapy were associated with a lower risk.

**Figure 2:**
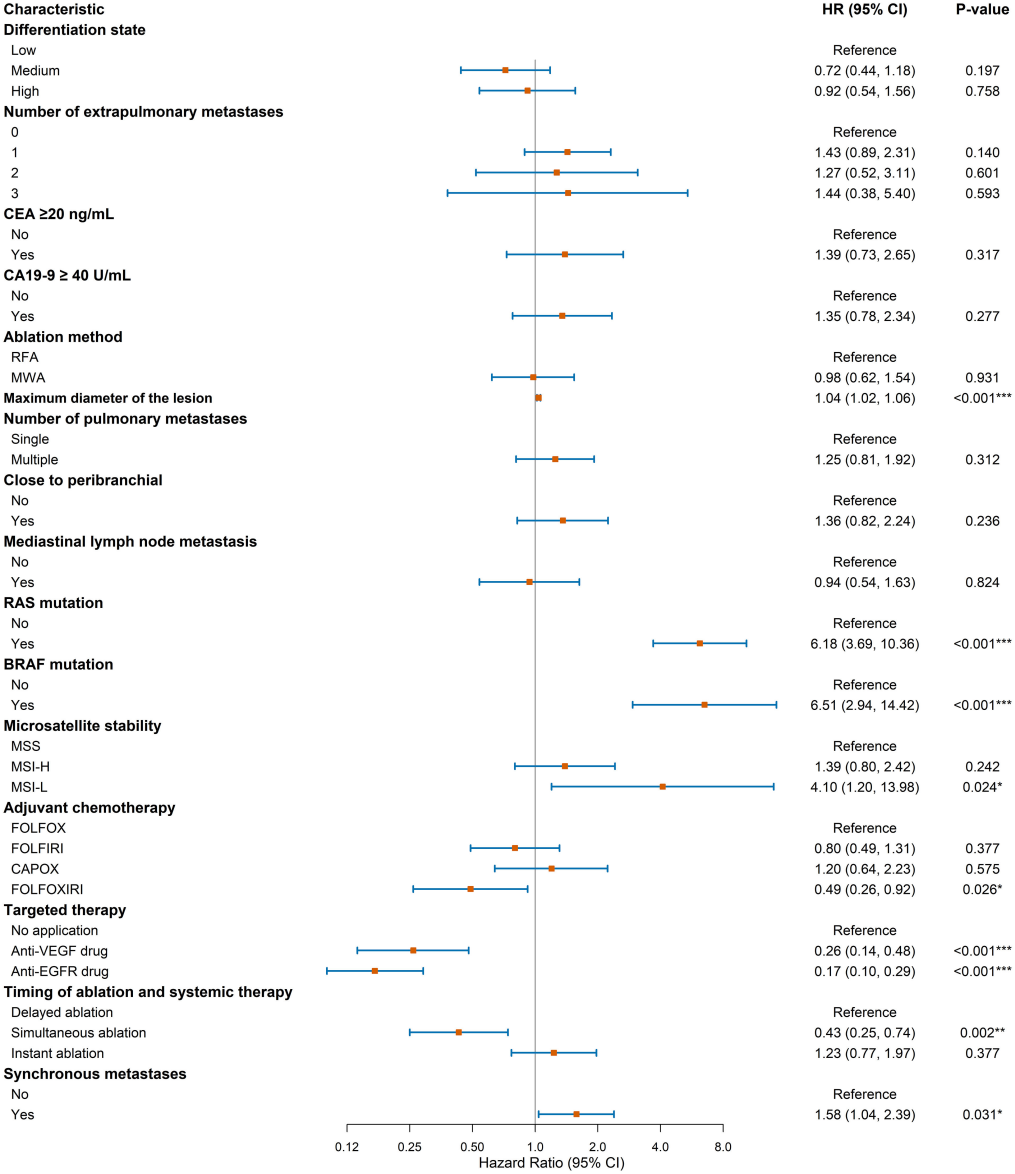
Forest plot of multivariable Cox regression analysis for overall survival *P<0.05; **P<0.01; ***P<0.001.

**Figure 3:**
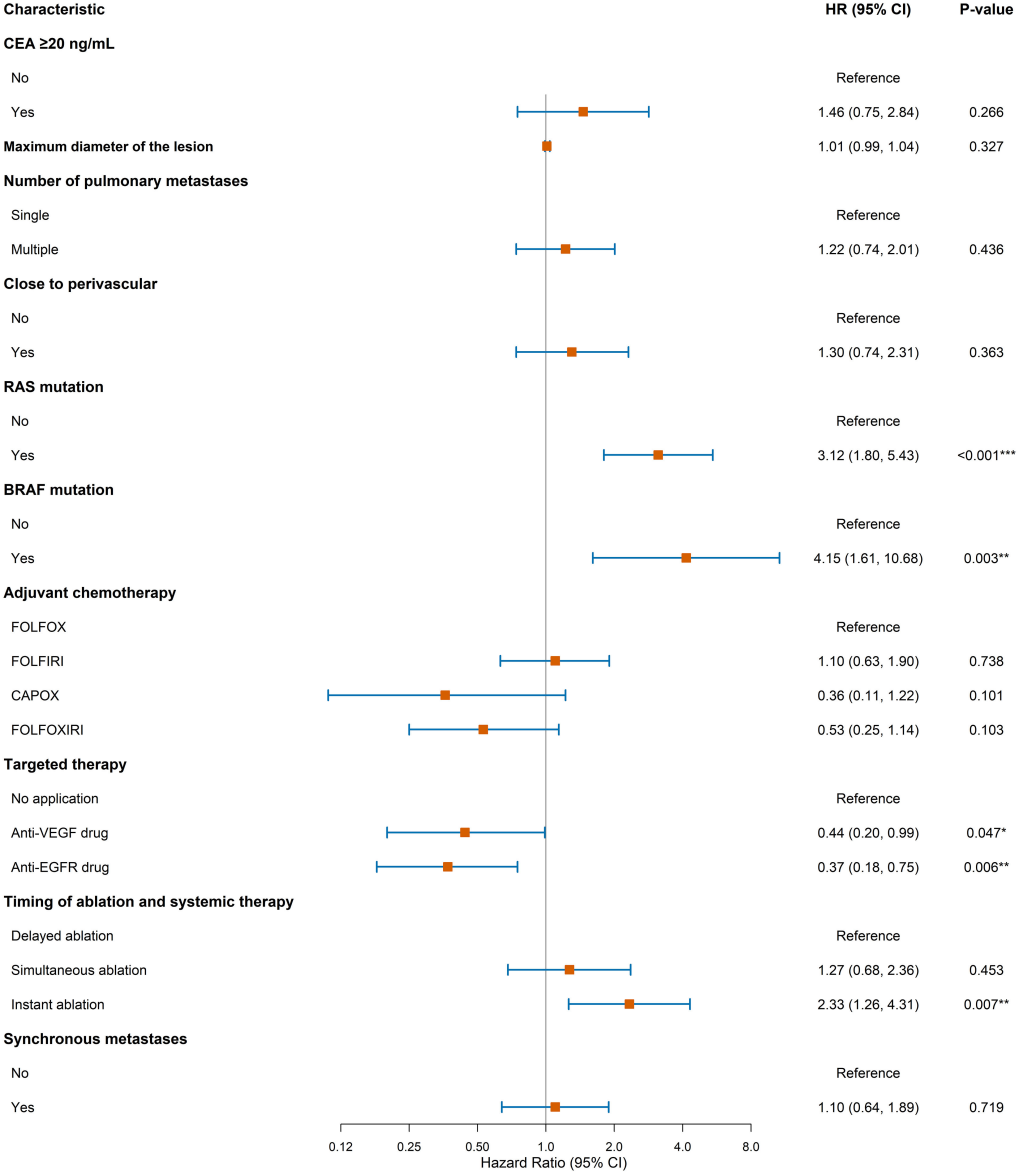
Forest plot of multivariable Cox regression analysis for local tumor progression-free survival Note: *P<0.05; **P<0.01; ***P<0.001.

Multivariable analysis further identified independent factors associated with each outcome. In the OS model, maximum lesion diameter (HR, 1.04; 95% CI, 1.02-1.06; P < 0.001), RAS mutation (HR, 6.18; 95% CI, 3.69-10.36; P < 0.001), BRAF mutation (HR, 6.51; 95% CI, 2.94-14.42; P < 0.001), MSI-L (HR, 4.10; 95% CI, 1.20-13.98; P = 0.024), and synchronous metastasis (HR, 1.58; 95% CI, 1.04-2.39; P = 0.031) were independent adverse factors, whereas FOLFOXIRI (HR, 0.49; 95% CI, 0.26-0.92; P = 0.026), anti-VEGF therapy (HR, 0.26; 95% CI, 0.14-0.48; P < 0.001), anti-EGFR therapy (HR, 0.17; 95% CI, 0.10-0.29; P < 0.001), and simultaneous ablation (HR, 0.43; 95% CI, 0.25-0.74; P = 0.002) were independent protective factors (**Figure 2**). In the PFS model, T3-T4 disease (HR, 1.71; 95% CI, 1.07-2.73; P = 0.026), multiple pulmonary metastases (HR, 1.40; 95% CI, 1.01-1.92; P = 0.042), RAS mutation (HR, 2.06; 95% CI, 1.49-2.84; P < 0.001), and MSI-L (HR, 2.85; 95% CI, 1.19-6.87; P = 0.019) remained significant (**Supplementary Figure 1**). In the LTPFS model, RAS mutation (HR, 3.12; 95% CI, 1.80-5.43; P < 0.001), BRAF mutation (HR, 4.15; 95% CI, 1.61-10.68; P = 0.003), and instant ablation (HR, 2.33; 95% CI, 1.26-4.31; P = 0.007) were independent adverse factors, whereas anti-VEGF therapy (HR, 0.44; 95% CI, 0.20-0.99; P = 0.047) and anti-EGFR therapy (HR, 0.37; 95% CI, 0.18-0.75; P = 0.006) were independent protective factors (**Figure 3**).

### Survival outcomes according to the timing of ablation and systemic therapy

After patients were grouped according to the timing of ablation relative to systemic therapy, IPTW within the ATE framework was used for adjustment. Propensity scores were estimated using a multinomial logistic model that included 17 baseline covariates. Before weighting, imbalances were observed in variables such as vessel proximity, mediastinal lymph node metastasis, and synchronous metastasis; after weighting, the absolute SMDs for all covariates were reduced to less than 0.10, indicating substantially improved comparability among the groups (**Supplementary Table 4**; **Supplementary Figures 2**, **S3**).

**Figure 4:**
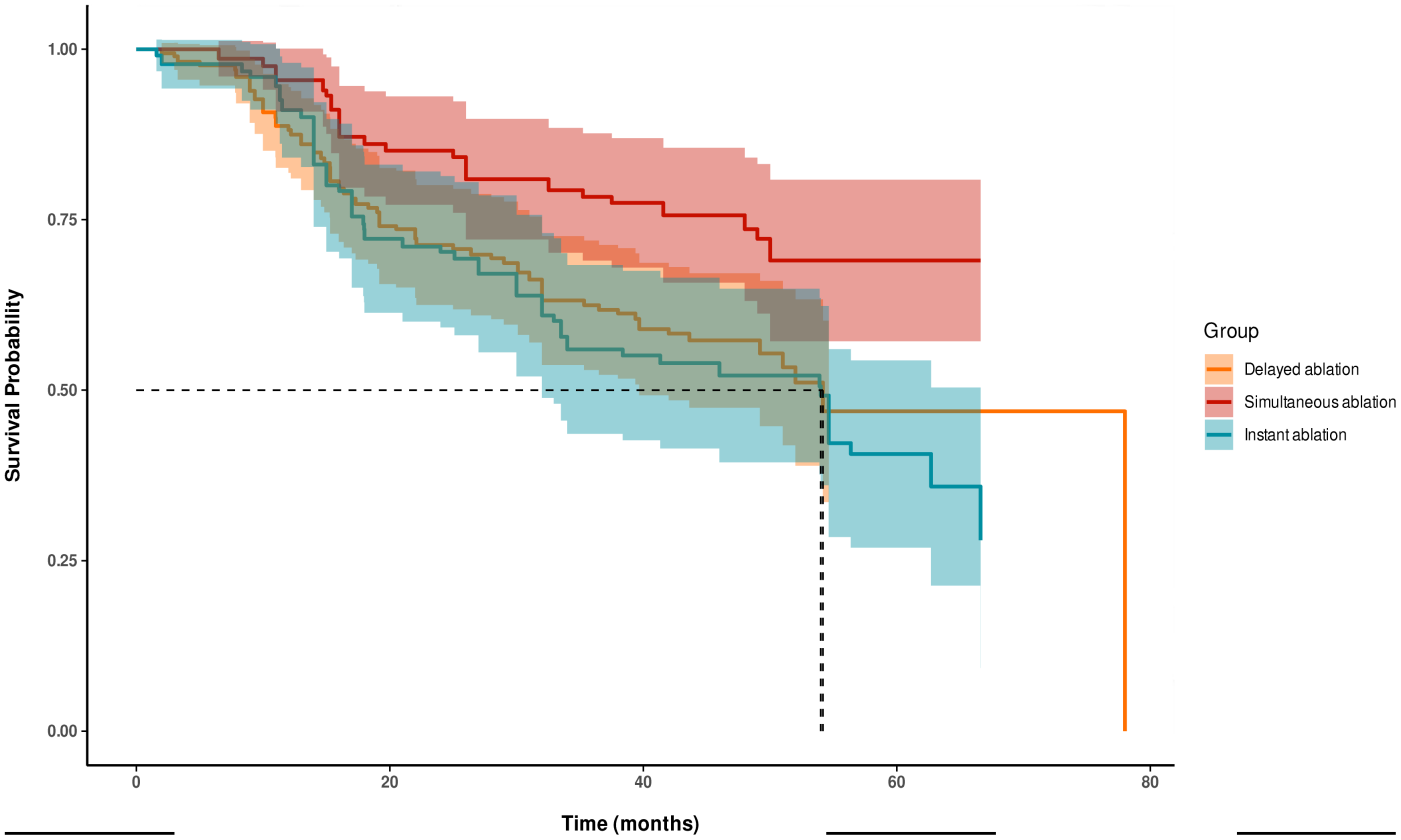
IPTW-weighted Kaplan-Meier curves for overall survival according to the timing of ablation relative to systemic therapy A number-at-risk table is not shown because the IPTW-adjusted curves represent a weighted pseudo-population rather than raw patient counts; displaying weighted, non-integer numbers at risk may be misleading.

After IPTW adjustment, simultaneous ablation was associated with a lower risk of death than delayed ablation (HR, 0.51; 95% CI, 0.27-0.96; P = 0.037), whereas instant ablation did not differ significantly from delayed ablation (HR, 1.16; 95% CI, 0.79-1.71; P = 0.447) (**Table 2**; **Figure 4**). The 12-, 36-, and 60-month OS rates in the simultaneous-ablation group were 95.5%, 78.3%, and 69.0%, respectively, all of which were higher than those in the delayed- and instant-ablation groups (**Supplementary Table 5**). For PFS, simultaneous ablation showed a trend toward improvement compared with delayed ablation (HR, 0.75; 95% CI, 0.55-1.03; P = 0.076), whereas instant ablation was not significantly different from delayed ablation (HR, 0.96; 95% CI, 0.65-1.41; P = 0.836); the median PFS in the simultaneous-ablation group was 24.0 months, longer than that in the delayed- and instant-ablation groups (**Table 2**; **Supplementary Table S6**; **Supplementary Figure 4**). For LTPFS, simultaneous ablation did not differ significantly from delayed ablation (HR, 1.39; 95% CI, 0.67-2.92; P = 0.378), whereas instant ablation was associated with a higher risk of local progression (HR, 2.62; 95% CI, 1.28-5.37; P = 0.008). The 24-month LTPFS rate was lowest in the instant-ablation group (**Table 2**; **Supplementary Table S7**; **Supplementary Figure 5**).

**Table 2:** Comparison of hazard ratios among different groups at the three endpoints after IPTW- weighted treatment.

Comparison	HR^1^	95% CI	p value
OS			
Simultaneous ablation vs delayed ablation	0.51	(0.27, 0.96)	0.037
Instant ablation vs delayed ablation	1.16	(0.79, 1.71)	0.447
LTPFS			
Simultaneous ablation vs delayed ablation	1.39	(0.67, 2.92)	0.378
Instant ablation vs delayed ablation	2.62	(1.28, 5.37)	0.008
PFS			
Simultaneous ablation vs delayed ablation	0.75	(0.55, 1.03)	0.076
Instant ablation vs delayed ablation	0.96	(0.65, 1.41)	0.836

IPTW, Inverse Probability of Treatment Weighting; OS, Overall survival; LTPFS, Local Tumor Progression- free Survival; PFS, Progression- free Survival; HR, Hazard Ratio; CI, Confidence Interval.

^1^ Variance estimation: Bootstrap, R = 500. n = 289.

A competing-risk analysis was further performed for local tumor progression, with death before local progression treated as a competing event. Overall, 71 local tumor progression events and 76 deaths before local progression were observed. In the unweighted Fine-Gray model, instant ablation remained associated with a higher cumulative incidence of local tumor progression compared with delayed ablation (SHR, 2.19; bootstrap 95% CI, 1.20-3.97; P = 0.010), whereas simultaneous ablation was not significantly different from delayed ablation (SHR, 1.59; bootstrap 95% CI, 0.85-2.96; P = 0.145). In the IPTW-weighted Fine-Gray model, instant ablation remained significantly associated with local tumor progression (SHR, 2.91; bootstrap 95% CI, 1.49-5.67; P = 0.002), whereas simultaneous ablation did not reach statistical significance (SHR, 1.94; bootstrap 95% CI, 0.97-3.89; P = 0.060) (**Supplementary Table 8**). The IPTW-weighted 24-month cumulative incidence of local tumor progression was 11.0%, 20.8%, and 31.5% in the delayed-, simultaneous-, and instant-ablation groups, respectively (**Supplementary Figure 6**).

### Adverse events

Among thermal ablation-related adverse events (AEs), pneumothorax was the most common and occurred in 118 patients. The incidence of pneumothorax did not differ significantly among the delayed-, simultaneous-, and instant-ablation groups (P = 0.093; chi-square = 4.74). Among pneumothorax events, 84.7% required no intervention or observation only, 9.3% were managed with needle aspiration, and 5.9% required closed thoracic drainage, with a median drainage duration of 4 days. In addition, pleural effusion occurred in 35 patients, and hemoptysis or blood-streaked sputum occurred in 50 patients. Other AEs included chest or abdominal pain in 54 patients, nausea or vomiting in 3, fever in 41, and pneumonia in 2. All AEs resolved after appropriate symptomatic and supportive treatment, and no permanent sequelae or treatment-related deaths were observed.

## Discussion

After IPTW adjustment, this study found that simultaneous ablation was associated with better overall survival than delayed or instant ablation and showed a trend toward improved PFS, whereas instant ablation was associated with a higher risk of local tumor progression. The central question was not whether ablation should be performed, but when ablation should be integrated into a comprehensive treatment strategy, which distinguishes this study from prior work.

In recent years, the management of colorectal lung metastases has shifted from isolated local treatment toward systemic therapy combined with local consolidation. Lee et al. analyzed 1, 143 patients and found that adding local ablative therapy to standard systemic therapy was associated with improved long-term survival (10). Karam et al. compared thermal ablation with surgery and reported comparable long-term outcomes between the two local approaches in appropriately selected patients (16). A network meta-analysis by Chierici et al. further showed that multimodal treatment yielded better survival outcomes than single-modality treatment (11). These findings are consistent with the present results and suggest that the timing of local therapy within the overall treatment plan may influence long-term outcomes.

In the present study, the advantage of the simultaneous-ablation strategy was observed mainly for OS rather than LTPFS alone. This distinction is clinically important. It suggests that the benefit of a synchronous strategy may reflect broader disease-course modification rather than improvement in local control of a single lesion alone. Systemic therapy can continuously suppress micrometastatic and occult extrapulmonary disease, whereas ablation can promptly eradicate radiographically visible lesions. Delivering both treatments within the same therapeutic window may shorten the duration of visible tumor burden and reduce gaps between local and systemic therapy. Reviews and guidelines on oligometastatic colorectal cancer similarly emphasize that systemic and local therapies should be integrated within an MDT framework rather than delivered as disconnected interventions (4, 17–19). From this perspective, the OS advantage associated with the synchronous strategy has a plausible clinical rationale.

Ichinose et al. proposed that delaying the decision to perform local treatment for pulmonary metastases may help identify patients with early rapid progression and thereby serve as a form of biologic selection (20). This concept remains clinically relevant. However, the poorer OS was observed in the delayed-ablation group than in the simultaneous-ablation group in our result. One possible explanation is that patients who have already entered the window for local therapy and can continue systemic treatment, prolonged waiting may not enhance the effect of local therapy itself and may instead extend the period during which visible disease remains untreated.

Although primary and secondary technical success rates were high, we observed worse LTPFS in the instant-ablation group. Meanwhile, this group had a higher baseline proportion of lesions adjacent to vessels. Previous studies have shown that local control after ablation of pulmonary metastases depends largely on whether an adequate ablation margin is achieved (18, 21, 22). In a study of cryoablation for colorectal lung metastases, Mohn et al. demonstrated a direct association between the three-dimensional minimal ablation margin and local control (23). Hong et al. also found, in a cone-beam CT-guided radiofrequency ablation cohort, that local tumor progression remained an important factor affecting long-term outcomes (24). In addition, local progression after thermal ablation is closely related to lesion location, particularly for tumors adjacent to vessels or bronchi, where heat-sink effects and irregular ablation geometry may reduce effective tumor coverage (25). Therefore, even when the measured minimal margin appeared adequate, local control may still have been affected by nonuniform circumferential margins or microscopic extension beyond the visible tumor boundary. The poorer LTPFS in the instant-ablation group may reflect greater difficulty in achieving complete local ablation, rather than a simple effect of treatment sequence alone.

Notably, the Cox analyses indicated that treatment timing was not the only determinant of outcome. RAS/BRAF status was associated not only with survival outcomes but also with local control. This finding is consistent with recent studies linking molecular features to the efficacy of local treatment. In a prospective study of radiofrequency ablation for colorectal liver metastases, Wang et al. showed that RAS mutation was associated with a higher risk of local progression and worse OS (26). Martin-Cullell et al. similarly observed that RAS/BRAF status was closely associated with outcomes after pulmonary metastasectomy (27). Patients with RAS wild-type disease, particularly those with left-sided primary tumors, are more likely to benefit from anti-EGFR therapy, whereas patients with RAS-mutant disease generally do not derive the same benefit (4, 28–30). BRAF mutation represents a molecular subtype with poor prognosis, and current treatment strategies increasingly emphasize targeted integration of the BRAF-EGFR axis (4, 31, 32). These observations suggest that treatment sequencing should be optimized on the basis of tumor biology and should not be interpreted independently of tumor burden and molecular features. In addition, MSI-H/dMMR is one of the clearest biomarkers of benefit from immunotherapy in metastatic colorectal cancer (33–35). KEYNOTE-177 demonstrated durable benefit with first-line pembrolizumab in patients with MSI-H/dMMR metastatic colorectal cancer (36). The CheckMate 8HW trial further showed improved disease control with nivolumab plus ipilimumab in this population (37). Thus, MSI status is relevant not only for prognostic assessment but also for treatment selection. For patients with MSI-H/dMMR disease, the timing of local therapy should be reconsidered within a PD-1-based systemic treatment framework. In the present study, MSI-L was associated with poorer OS and PFS; however, this finding should be interpreted cautiously because the number of MSI-L cases was limited. Larger prospective studies are needed to validate this finding.

This study has several limitations. First, because of its retrospective multicenter design, treatment sequence was determined by routine clinical decision-making rather than randomization. The choice of delayed, synchronous, or instant ablation may have reflected tumor burden, lesion location, response to systemic therapy, patient condition, and local institutional practice. IPTW helped reduce measured baseline imbalance, but it could not remove unmeasured confounding. Second, systemic therapy was not completely uniform during the study period. From 2015 to 2023, treatment strategies for metastatic colorectal cancer continued to evolve, and detailed information on treatment line, treatment duration, number of cycles, maintenance therapy, and later-line agents was not consistently available. These factors may have affected OS and PFS independently of ablation timing. Third, local control after ablation may have been influenced by technical factors that were only partly captured in this dataset. Ablation margins were assessed retrospectively on routine CT images, but detailed three-dimensional margin geometry, energy delivery, probe trajectory, operator selection, and heat-sink effects in perivascular lesions were not fully evaluated. Therefore, the poorer LTPFS observed in the instant-ablation group should be interpreted in the context of both treatment timing and lesion-level technical complexity. Finally, follow-up imaging was performed according to real-world practice rather than a uniform prospective protocol, and the number of local progression events was limited after stratification into three groups.

In conclusion, this cohort study of patients undergoing ablation for oligometastatic colorectal lung metastases systematically evaluated the association of treatment sequencing between local ablation and systemic therapy with survival outcomes and local control. The prognostic value of different sequencing strategies should be further validated in multicenter prospective cohorts or clinical trials, and the timing of ablation should be incorporated as an important variable in MDT-based comprehensive treatment planning.

## Data Availability

The raw data supporting the conclusions of this article will be made available by the authors, without undue reservation.
