# Supplementary material for: Outcomes following different thermal ablation strategies in patients with oligometastatic colorectal lung metastases
Source: Front Oncol. 2026 Jul 8;16:1902067. doi: 10.3389/fonc.2026.1902067 (PMC13388081; doi:10.3389/fonc.2026.1902067)
Supplement: Supplementary file 1 [file DataSheet1.docx]

**Outcomes Following Different Thermal Ablation Strategies in Patients with oligometastatic Colorectal Lung Metastases**

| \| **Table S1. Missing Data in The Pre-imputation Dataset** \| \| \| \| --- \| --- \| --- \| \| **Variable** \| **Missing n/N** \| **Missing %** \| \| **Differentiation state** \| 42/289 \| 14.5 \| \| **Number of extrapulmonary metastases** \| 14/289 \| 4.8 \| \| **CEA** \| 23/289 \| 7.9 \| \| **CA19-9** \| 44/289 \| 15.2 \| \| **Number of pulmonary metastases** \| 11/289 \| 3.8 \| \| **Close to perivascular** \| 1/289 \| 0.3 \| \| **Close to peribronchial** \| 10/289 \| 3.5 \|   Abbreviation: CEA, Carcinoembryonic Antigen; CA19-9, Carbohydrate Antigen 19-9  **Table S2. Ablation Margin Assessment in Three Groups** | | | | |
| --- | --- | --- | --- | --- | --- | --- | --- | --- | --- | --- | --- | --- | --- | --- | --- | --- | --- | --- | --- | --- | --- | --- | --- | --- | --- | --- | --- | --- | --- | --- | --- |
| **Characteristic** | **Delayed ablation** | **Simultaneous ablation** | **Instant ablation** | **p-value^1^** |
| Number of treated lesions | 217 | 147 | 149 | NA |
| Minimal ablation margin, median (IQR), mm | 6.7 | 7.0 | 7.5 | 0.234 |
| Adequate margin ≥5 mm, n (%) | 212 | 145 | 143 | 0.350 |
| Insufficient margin <5 mm, n (%) | 5 | 2 | 6 | 0.350 |
| Margin not assessable, n (%) | 0 | 0 | 0 | NA |

^1^Pearson's Chi-squared test; Fisher exact test; Kruskal-Wallis rank sum test

| **Table S3. Univariable COX Analysis of Three Endpoints** | | | | | | | | | |
| --- | --- | --- | --- | --- | --- | --- | --- | --- | --- |
| **Characteristic** | **OS** | | | **LTPFS** | | | **PFS** | | |
|  | **HR** | **95% CI** | **p-value^1^** | **HR** | **95% CI** | **p-value^1^** | **HR** | **95% CI** | **p-value^1^** |
| **Sex** |  |  |  |  |  |  |  |  |  |
| Male | — | — |  | — | — |  | — | — |  |
| Female | 1.02 | 0.71, 1.47 | 0.908 | 1.24 | 0.78, 1.98 | 0.360 | 0.90 | 0.68, 1.19 | 0.452 |
| **Age** |  |  |  |  |  |  |  |  |  |
| ＜ 60 y | — | — |  | — | — |  | — | — |  |
| ≥ 60 y | 0.94 | 0.66, 1.35 | 0.754 | 0.88 | 0.55, 1.40 | 0.591 | 1.22 | 0.93, 1.60 | 0.152 |
| **Primary tumor site** |  |  |  |  |  |  |  |  |  |
| Rectum | — | — |  | — | — |  | — | — |  |
| Colon | 0.87 | 0.60, 1.26 | 0.458 | 1.04 | 0.64, 1.68 | 0.882 | 1.13 | 0.85, 1.50 | 0.393 |
| **Differentiation state** |  |  |  |  |  |  |  |  |  |
| Low | — | — |  | — | — |  | — | — |  |
| Medium | 0.63 | 0.41, 0.97 | 0.037 | 0.80 | 0.45, 1.40 | 0.434 | 0.73 | 0.52, 1.01 | 0.055 |
| High | 0.72 | 0.45, 1.15 | 0.172 | 0.74 | 0.39, 1.40 | 0.359 | 0.66 | 0.46, 0.95 | 0.027* |
| **T designation** |  |  |  |  |  |  |  |  |  |
| T1-T2 | — | — |  | — | — |  | — | — |  |
| T3-T4 | 1.21 | 0.72, 2.06 | 0.473 | 0.92 | 0.49, 1.71 | 0.794 | 1.90 | 1.23, 2.94 | 0.004** |
| **Number of extrapulmonary metastases** |  |  |  |  |  |  |  |  |  |
| 0 | — | — |  | — | — |  | — | — |  |
| 1 | 1.56 | 1.05, 2.34 | 0.030 | 0.96 | 0.54, 1.71 | 0.892 | 1.52 | 1.11, 2.08 | 0.009** |
| 2 | 1.14 | 0.50, 2.62 | 0.757 | 0.51 | 0.12, 2.09 | 0.349 | 3.20 | 1.84, 5.58 | <0.001*** |
| 3 | 2.99 | 0.94, 9.52 | 0.064 | 1.10 | 0.15, 7.94 | 0.927 | 2.01 | 0.74, 5.45 | 0.169 |
| **CEA ≥20 ng/mL** |  |  |  |  |  |  |  |  |  |
| No | — | — |  | — | — |  | — | — |  |
| Yes | 1.56 | 0.95, 2.55 | 0.077 | 1.68 | 0.90, 3.13 | 0.102 | 1.25 | 0.84, 1.86 | 0.279 |
| **CA199 ≥ 40 U/mL** |  |  |  |  |  |  |  |  |  |
| No | — | — |  | — | — |  | — | — |  |
| Yes | 1.98 | 1.31, 2.98 | 0.001 | 1.20 | 0.66, 2.18 | 0.561 | 1.29 | 0.91, 1.81 | 0.148 |
| **Ablation method** |  |  |  |  |  |  |  |  |  |
| RFA | — | — |  | — | — |  | — | — |  |
| MWA | 0.66 | 0.46, 0.95 | 0.024 | 1.21 | 0.75, 1.97 | 0.434 | 0.69 | 0.53, 0.91 | 0.008** |
| **Maximum diameter of the lesion** | 1.05 | 1.03, 1.07 | <0.001 | 1.02 | 1.00, 1.04 | 0.081 | 1.03 | 1.02, 1.05 | <0.001*** |
| **Number of pulmonary metastases** |  |  |  |  |  |  |  |  |  |
| Single | — | — |  | — | — |  | — | — |  |
| Multiple | 2.03 | 1.40, 2.95 | <0.001 | 1.43 | 0.90, 2.28 | 0.132 | 1.80 | 1.37, 2.37 | <0.001*** |
| **Close to perivascular** |  |  |  |  |  |  |  |  |  |
| No | — | — |  | — | — |  | — | — |  |
| Yes | 1.18 | 0.75, 1.85 | 0.471 | 1.51 | 0.87, 2.61 | 0.139 | 0.97 | 0.69, 1.37 | 0.878 |
| **Close to peribronchial** |  |  |  |  |  |  |  |  |  |
| No | — | — |  | — | — |  | — | — |  |
| Yes | 1.53 | 0.97, 2.42 | 0.066 | 1.11 | 0.57, 2.16 | 0.764 | 1.23 | 0.85, 1.77 | 0.274 |
| **Hilar lymph node metastasis** |  |  |  |  |  |  |  |  |  |
| No | — | — |  | — | — |  | — | — |  |
| Yes | 1.31 | 0.82, 2.11 | 0.258 | 0.85 | 0.42, 1.71 | 0.649 | 0.83 | 0.56, 1.23 | 0.356 |
| **Mediastinal lymph node metastasis** |  |  |  |  |  |  |  |  |  |
| No | — | — |  | — | — |  | — | — |  |
| Yes | 2.50 | 1.61, 3.88 | <0.001 | 1.12 | 0.53, 2.33 | 0.771 | 2.17 | 1.51, 3.11 | <0.001*** |
| **RAS mutation** |  |  |  |  |  |  |  |  |  |
| No | — | — |  | — | — |  | — | — |  |
| Yes | 3.33 | 2.28, 4.88 | <0.001 | 2.32 | 1.44, 3.73 | <0.001*** | 1.96 | 1.49, 2.59 | <0.001*** |
| **BRAF mutation** |  |  |  |  |  |  |  |  |  |
| No | — | — |  | — | — |  | — | — |  |
| Yes | 2.24 | 1.30, 3.86 | 0.004 | 1.73 | 0.79, 3.78 | 0.171 | 1.58 | 1.01, 2.46 | 0.043* |
| **Microsatellite stability** |  |  |  |  |  |  |  |  |  |
| MSS | — | — |  | — | — |  | — | — |  |
| MSI-H | 2.06 | 1.31, 3.23 | 0.002 | 1.11 | 0.55, 2.24 | 0.762 | 1.47 | 1.01, 2.14 | 0.043* |
| MSI-L | 1.33 | 0.42, 4.20 | 0.629 | NA | NA | NA | 1.59 | 0.70, 3.59 | 0.265 |
| **Adjuvant chemotherapy** |  |  |  |  |  |  |  |  |  |
| FOLFOX | — | — |  | — | — |  | — | — |  |
| FOLFIRI | 0.63 | 0.41, 0.97 | 0.035 | 0.97 | 0.57, 1.63 | 0.895 | 0.67 | 0.48, 0.92 | 0.015* |
| CAPOX | 1.13 | 0.64, 1.99 | 0.667 | 0.36 | 0.11, 1.18 | 0.091 | 0.79 | 0.50, 1.25 | 0.314 |
| FOLFOXIRI | 0.47 | 0.27, 0.82 | 0.008 | 0.61 | 0.30, 1.22 | 0.164 | 0.79 | 0.54, 1.15 | 0.218 |
| **Targeted therapy** |  |  |  |  |  |  |  |  |  |
| No application | — | — |  | — | — |  | — | — |  |
| Anti-VEGF drug | 0.15 | 0.09, 0.25 | <0.001 | 0.33 | 0.16, 0.68 | 0.002** | 0.40 | 0.26, 0.62 | <0.001*** |
| Anti-EGFR drug | 0.22 | 0.14, 0.35 | <0.001 | 0.38 | 0.20, 0.74 | 0.004** | 0.69 | 0.46, 1.02 | 0.065 |
| **Timing of ablation and systemic therapy** |  |  |  |  |  |  |  |  |  |
| Delayed ablation | — | — |  | — | — |  | — | — |  |
| Simultaneous ablation | 0.46 | 0.28, 0.76 | 0.002 | 1.33 | 0.73, 2.44 | 0.352 | 0.71 | 0.51, 0.98 | 0.039* |
| Instant ablation | 0.99 | 0.66, 1.48 | 0.946 | 2.03 | 1.15, 3.60 | 0.015* | 0.82 | 0.59, 1.14 | 0.248 |
| **Synchronous metastases** |  |  |  |  |  |  |  |  |  |
| No | — | — |  | — | — |  | — | — |  |
| Yes | 2.18 | 1.50, 3.18 | <0.001 | 1.54 | 0.92, 2.57 | 0.097 | 1.50 | 1.11, 2.03 | 0.008** |

Abbreviations: OS, Overall survival; LTPFS, Local Tumor Progression- free Survival; PFS, Progression- free Survival; CI = Confidence Interval, HR = Hazard Ratio; CEA, Carcinoembryonic Antigen; CA19-9, Carbohydrate Antigen 19-9; RFA, Radiofrequency Ablation; MWA, Microwave Ablation; RAS, Rat Sarcoma viral oncogene homolog; BRAF, v-Raf murine sarcoma viral oncogene homolog B; MSS, Microsatellite Stable; MSI-H, Microsatellite Instability-High; MSI-L, Microsatellite Instability-Low; FOLFOX, Folinic acid (Leucovorin) + Fluorouracil (5-FU) + Oxaliplatin; FOLFIRI, Folinic acid (Leucovorin) + Fluorouracil (5-FU) + Irinotecan; CAPOX, Capecitabine + Oxaliplatin; FOLFOXIRI, Folinic acid + Fluorouracil + Oxaliplatin + Irinotecan; EGFR, Epidermal Growth Factor Receptor; VEGF, Vascular Endothelial Growth Factor.

^1^*p<0.05; **p<0.01; ***p<0.001

| **Table S4. Baseline Covariates Before and After Inverse Probability of Treatment Weighting** | | | | | | | | |
| --- | --- | --- | --- | --- | --- | --- | --- | --- |
|  | **Unweighted** | | | | **Weighted** | | | |
|  | **Delayed ablation**  n = 118 | **Simultaneous ablation**  n = 87 | **Instant ablation**  n = 84 | **SMD** | **Delayed ablation** | **Simultaneous ablation** | **Instant ablation** | **SMD** |
| **Sex, n (%)** |  |  |  |  |  |  |  |  |
| Male | 67 (57%) | 47 (54%) | 52 (62%) | -0.079 | 57% | 56% | 56% | -0.015 |
| Female | 51 (43%) | 40 (46%) | 32 (38%) | 0.079 | 43% | 44% | 44% | 0.015 |
| **Age, n (%)** |  |  |  |  |  |  |  |  |
| ＜ 60 y | 54 (46%) | 46 (53%) | 38 (45%) | -0.076 | 46% | 46% | 49% | -0.031 |
| ≥ 60 y | 64 (54%) | 41 (47%) | 46 (55%) | 0.076 | 54% | 54% | 51% | 0.031 |
| **Primary tumor site, n (%)** |  |  |  |  |  |  |  |  |
| Rectum | 42 (36%) | 39 (45%) | 32 (38%) | -0.092 | 40% | 41% | 40% | -0.013 |
| Colon | 76 (64%) | 48 (55%) | 52 (62%) | 0.092 | 60% | 59% | 60% | 0.013 |
| **Differentiation state, n (%)** |  |  |  |  |  |  |  |  |
| Low | 28 (24%) | 23 (26%) | 24 (29%) | 0.048 | 26% | 26% | 33% | 0.068 |
| Medium | 52 (44%) | 45 (52%) | 37 (44%) | 0.077 | 48% | 49% | 43% | 0.053 |
| High | 38 (32%) | 19 (22%) | 23 (27%) | 0.104 | 26% | 25% | 24% | 0.024 |
| **T designation, n (%)** |  |  |  |  |  |  |  |  |
| T1-T2 | 19 (16%) | 12 (14%) | 14 (17%) | -0.029 | 14% | 13% | 13% | -0.011 |
| T3-T4 | 99 (84%) | 75 (86%) | 70 (83%) | 0.029 | 86% | 87% | 87% | 0.011 |
| **Number of extrapulmonary metastases, n (%)** |  |  |  |  |  |  |  |  |
| 0 | 86 (73%) | 60 (69%) | 59 (70%) | 0.039 | 70% | 72% | 72% | 0.022 |
| 1 | 23 (19%) | 22 (25%) | 21 (25%) | 0.058 | 24% | 22% | 22% | 0.022 |
| 2 | 8 (6.8%) | 4 (4.6%) | 2 (2.4%) | 0.044 | 4.9% | 5.1% | 4.2% | 0.008 |
| 3 | 1 (0.8%) | 1 (1.1%) | 2 (2.4%) | 0.015 | 1.2% | 1.4% | 1.6% | 0.004 |
| **CEA≥20 ng/mL, n (%)** |  |  |  |  |  |  |  |  |
| No | 101 (86%) | 74 (85%) | 77 (92%) | -0.066 | 87% | 88% | 88% | -0.010 |
| Yes | 17 (14%) | 13 (15%) | 7 (8.3%) | 0.066 | 13% | 12% | 12% | 0.010 |
| **CA19-9≥40 U/mL, n (%)** |  |  |  |  |  |  |  |  |
| No | 96 (81%) | 70 (80%) | 69 (82%) | -0.017 | 82% | 81% | 81% | -0.013 |
| Yes | 22 (19%) | 17 (20%) | 15 (18%) | 0.017 | 18% | 19% | 19% | 0.013 |
| **Number of**  **pulmonary metastases, n (%)** |  |  |  |  |  |  |  |  |
| Single | 57 (48%) | 49 (56%) | 44 (52%) | -0.080 | 52% | 52% | 56% | -0.048 |
| Multiple | 61 (52%) | 38 (44%) | 40 (48%) | 0.080 | 48% | 48% | 44% | 0.048 |
| **Close to perivascular, n (%)** |  |  |  |  |  |  |  |  |
| No | 104 (88%) | 69 (79%) | 62 (74%) | -0.143 | 81% | 80% | 82% | -0.018 |
| Yes | 14 (12%) | 18 (21%) | 22 (26%) | 0.143 | 19% | 20% | 18% | 0.018 |
| **Close to peribronchial, n (%)** |  |  |  |  |  |  |  |  |
| No | 101 (86%) | 77 (89%) | 68 (81%) | -0.076 | 85% | 87% | 86% | -0.016 |
| Yes | 17 (14%) | 10 (11%) | 16 (19%) | 0.076 | 15% | 13% | 14% | 0.016 |
| **Hilar lymph node metastasis, n (%)** |  |  |  |  |  |  |  |  |
| No | 99 (84%) | 73 (84%) | 76 (90%) | -0.066 | 87% | 86% | 88% | -0.025 |
| Yes | 19 (16%) | 14 (16%) | 8 (9.5%) | 0.066 | 13% | 14% | 12% | 0.025 |
| **Mediastinal lymph node metastasis, n (%)** |  |  |  |  |  |  |  |  |
| No | 96 (81%) | 78 (90%) | 77 (92%) | -0.103 | 87% | 86% | 86% | -0.008 |
| Yes | 22 (19%) | 9 (10%) | 7 (8.3%) | 0.103 | 13% | 14% | 14% | 0.008 |
| **RAS mutation, n (%)** |  |  |  |  |  |  |  |  |
| No | 63 (53%) | 51 (59%) | 52 (62%) | -0.085 | 57% | 58% | 56% | -0.020 |
| Yes | 55 (47%) | 36 (41%) | 32 (38%) | 0.085 | 43% | 42% | 44% | 0.020 |
| **BRAF mutation, n (%)** |  |  |  |  |  |  |  |  |
| No | 105 (89%) | 81 (93%) | 77 (92%) | -0.041 | 92% | 91% | 93% | -0.020 |
| Yes | 13 (11%) | 6 (6.9%) | 7 (8.3%) | 0.041 | 8.5% | 8.8% | 6.8% | 0.020 |
| **Microsatellite stability, n (%)** |  |  |  |  |  |  |  |  |
| MSS | 99 (84%) | 75 (86%) | 69 (82%) | 0.041 | 84% | 85% | 83% | 0.016 |
| MSI-H | 17 (14%) | 9 (10%) | 13 (15%) | 0.051 | 14% | 13% | 15% | 0.017 |
| MSI-L | 2 (1.7%) | 3 (3.4%) | 2 (2.4%) | 0.018 | 2.2% | 2.1% | 2.0% | 0.002 |
| **Synchronous metastases, n (%)** |  |  |  |  |  |  |  |  |
| No | 92 (78%) | 67 (77%) | 57 (68%) | -0.101 | 77% | 75% | 78% | -0.025 |
| Yes | 26 (22%) | 20 (23%) | 27 (32%) | 0.101 | 23% | 25% | 22% | 0.025 |
| ^Abbreviation: SMD, Standardized Mean Difference; CEA,^ ^Carcinoembryonic Antigen; CA19-9, Carbohydrate Antigen 19-9; RAS, Rat Sarcoma viral oncogene homolog; BRAF, v-Raf murine sarcoma viral oncogene homolog B; MSS, Microsatellite Stable; MSI-H, Microsatellite Instability-High; MSI-L, Microsatellite Instability-Low.^ | | | | | | | | |

| Table S5. IPTW-weighted Survival Rate at Specified Time Points of Overall Survival | | | |
| --- | --- | --- | --- |
| **Group** | **Time (months)** | **Survival Rate** | **95% CI** |
| Delayed ablation | 12 | 88.1% | (81.9%, 94.4%) |
| Delayed ablation | 36 | 62.5% | (53.0%, 71.9%) |
| Delayed ablation | 60 | 46.9% | — |
| Simultaneous ablation | 12 | 95.5% | (90.9%, 100.1%) |
| Simultaneous ablation | 36 | 78.3% | (69.0%, 87.6%) |
| Simultaneous ablation | 60 | 69.0% | (57.2%, 80.8%) |
| Instant ablation | 12 | 91.1% | (84.1%, 98.0%) |
| Instant ablation | 36 | 56.0% | (43.6%, 68.3%) |
| Instant ablation | 60 | 40.6% | (26.9%, 54.4%) |

Abbreviation: IPTW, Inverse Probability of Treatment Weighting; CI, Confidence Interval.

| Table S6. IPTW-weighted Survival Rate at Specified Time Points of Progression- free Survival | | | |
| --- | --- | --- | --- |
| **Group** | **Time (months)** | **Survival Rate** | **95% CI** |
| Delayed ablation | 6 | 88.8% | (82.7%, 95.0%) |
| Delayed ablation | 12 | 58.5% | (49.1%, 68.0%) |
| Delayed ablation | 36 | 24.4% | (16.0%, 32.8%) |
| Simultaneous ablation | 6 | 93.2% | (87.6%, 98.8%) |
| Simultaneous ablation | 12 | 69.4% | (59.1%, 79.6%) |
| Simultaneous ablation | 36 | 40.6% | (29.5%, 51.7%) |
| Instant ablation | 6 | 85.0% | (76.5%, 93.5%) |
| Instant ablation | 12 | 62.1% | (50.8%, 73.4%) |
| Instant ablation | 36 | 23.2% | (13.0%, 33.4%) |

Abbreviation: IPTW, Inverse Probability of Treatment Weighting; CI, Confidence Interval.

| Table S7. IPTW-weighted Survival Rate at Specified Time Points of Local Tumor Progression- free Survival | | | |
| --- | --- | --- | --- |
| **Group** | **Time (months)** | **Survival Rate** | **95% CI** |
| Delayed ablation | 6 | 97.8% | (95.0%, 100.7%) |
| Delayed ablation | 12 | 93.2% | (88.1%, 98.2%) |
| Delayed ablation | 24 | 85.6% | (78.1%, 93.1%) |
| Simultaneous ablation | 6 | 97.7% | (94.3%, 101.0%) |
| Simultaneous ablation | 12 | 88.3% | (81.2%, 95.6%) |
| Simultaneous ablation | 24 | 81.8% | (72.9%, 90.6%) |
| Instant ablation | 6 | 92.7% | (86.4%, 99.0%) |
| Instant ablation | 12 | 84.5% | (75.7%, 93.2%) |
| Instant ablation | 24 | 66.6% | (54.4%, 78.7%) |

Abbreviation: IPTW, Inverse Probability of Treatment Weighting; CI, Confidence Interval.

| **Table S8. Fine-Gary Regression in Local Tumor Progression-free Survival** | | | | | |
| --- | --- | --- | --- | --- | --- |
| **Model** | **Comparison** | **SHR** | **Bootstrap 95% CI** | **Bootstrap P value** | **Valid bootstrap replicates** |
| Unweighted Fine-Gray | Simultaneous ablation vs Delayed ablation | 1.59 | 0.85-2.96 | 0.145 | 500 |
| Unweighted Fine-Gray | Instant ablation vs Delayed ablation | 2.19 | 1.20-3.97 | 0.010 | 500 |
| IPTW-weighted Fine-Gray | Simultaneous ablation vs Delayed ablation | 1.94 | 0.97-3.89 | 0.060 | 500 |
| IPTW-weighted Fine-Gray | Instant ablation vs Delayed ablation | 2.91 | 1.49-5.67 | 0.002 | 500 |

Abbreviations: SHR, subdistribution hazard ratio; CI, confidence interval; IPTW, inverse probability of treatment weighting; LTP, local tumor progression. Bootstrap confidence intervals and P values were calculated from patient-level bootstrap resampling (500 requested replicates). IPTW weights were fixed from the primary propensity-score model.


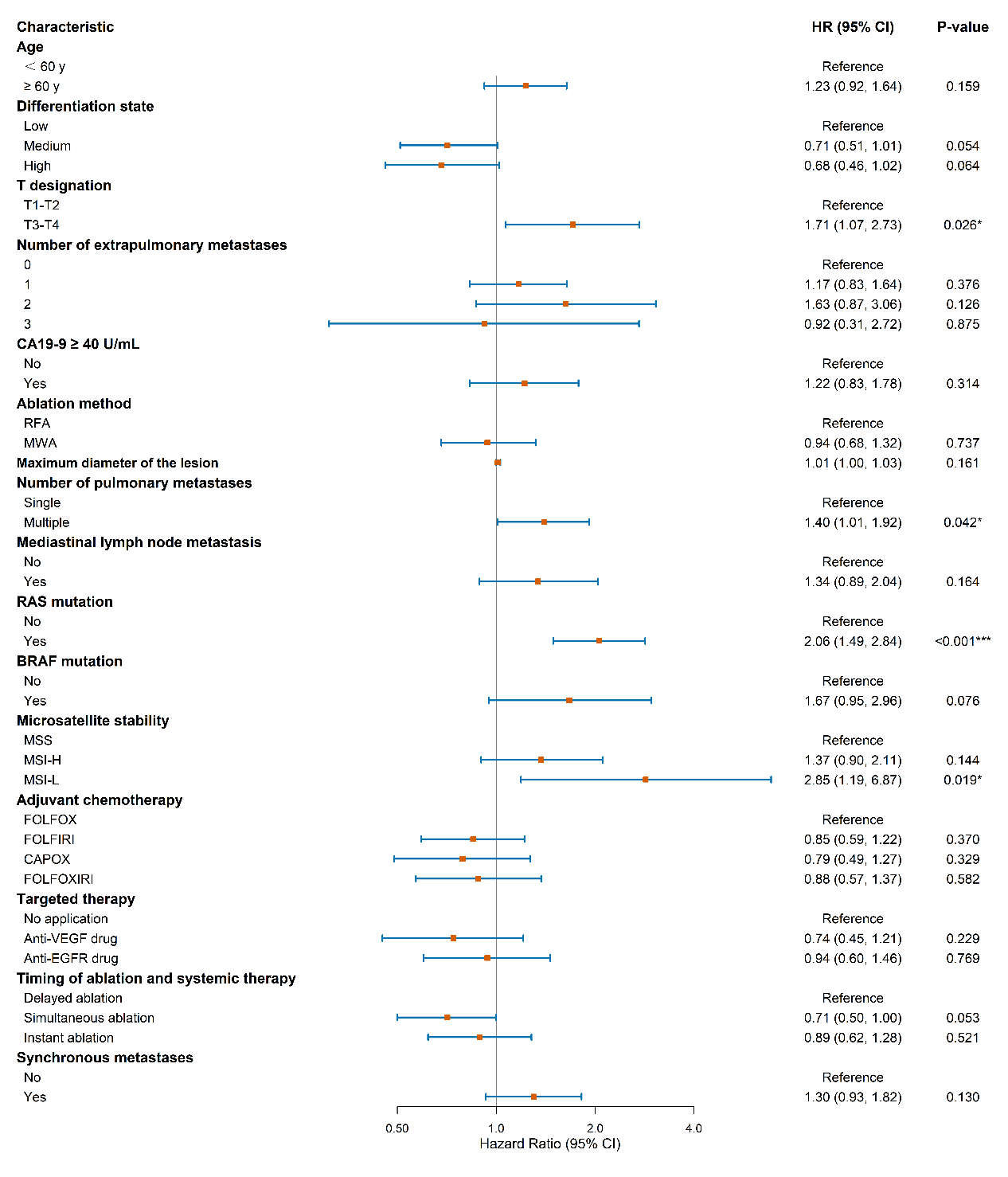


**Figure S1 Forest plot of multivariable Cox regression analysis for progression-free survival** Note: *P<0.05; **P<0.01; ***P<0.001.

**
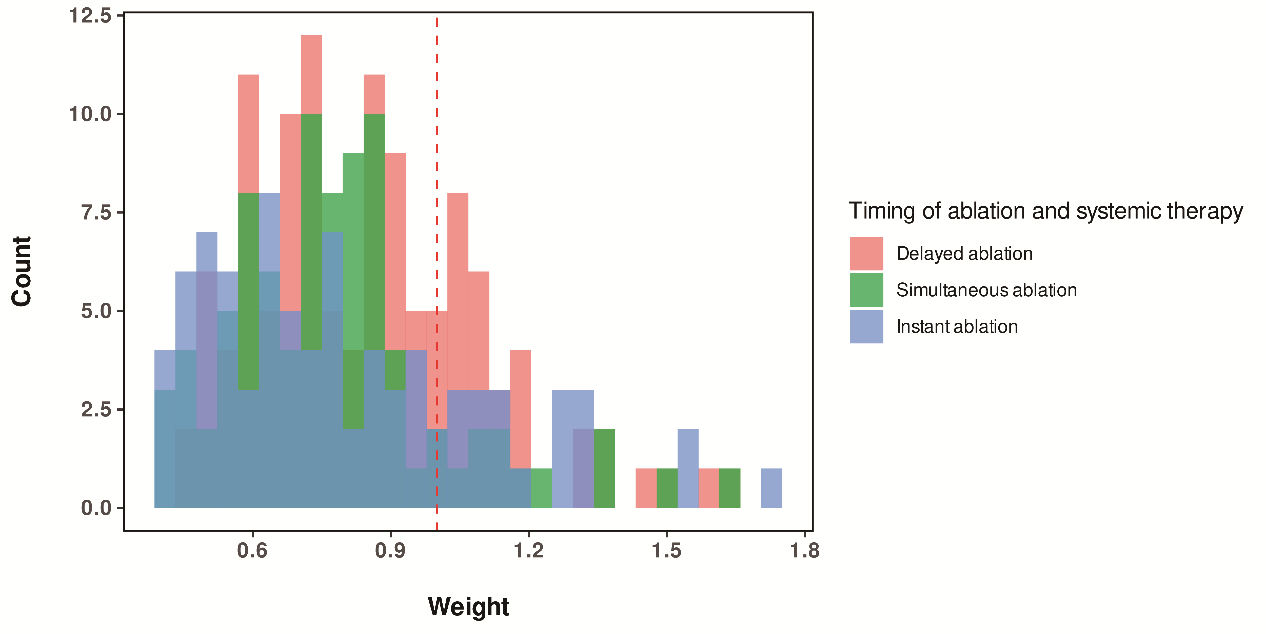
**

**Figure S2 Propensity score weighting weight distribution**


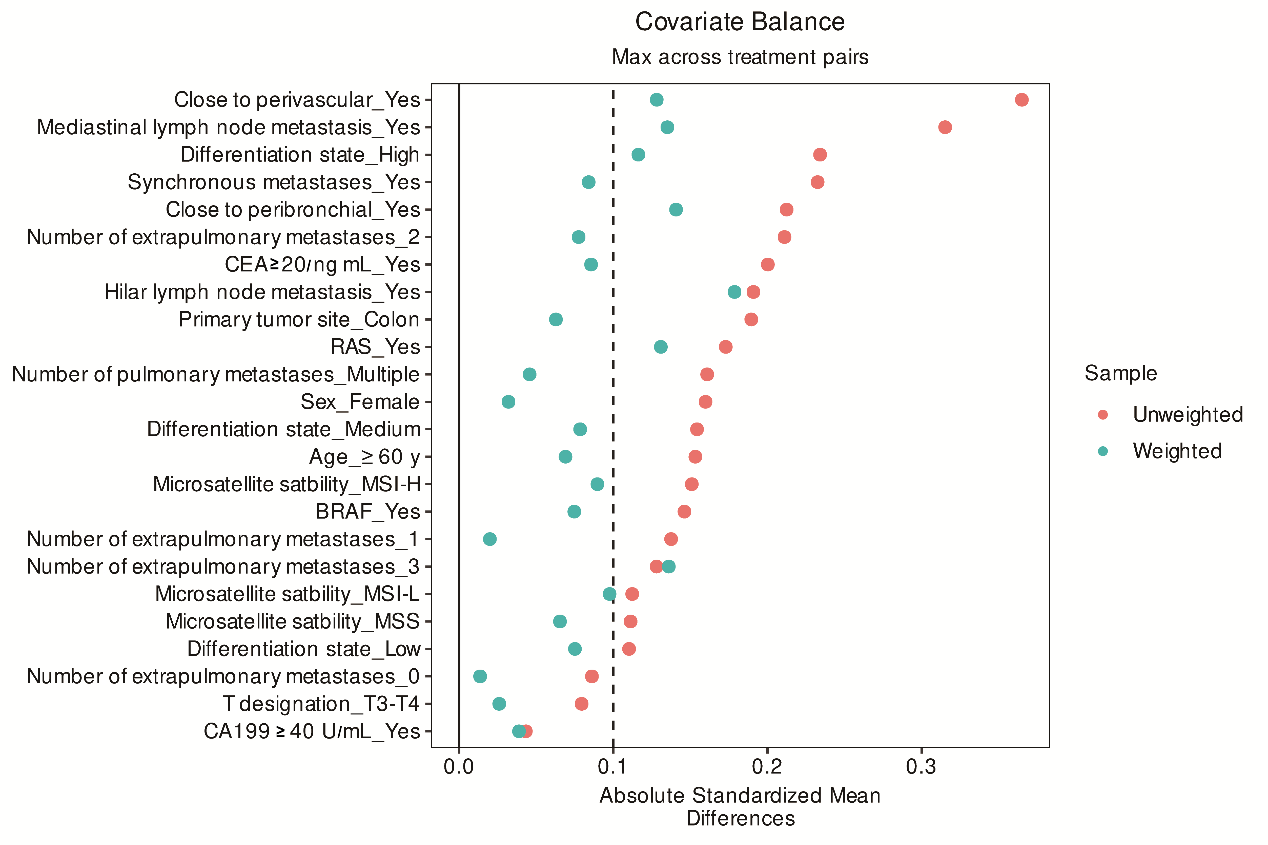


**Figure S3 Love plot of covariate balance before and after inverse probability of treatment weighting adjustment under the average treatment effect framework** Note: The Love plot displays the maximum absolute standardized mean difference (SMD) for each baseline covariate across pairwise comparisons among the delayed-ablation, simultaneous ablation, and instant-ablation groups before and after inverse probability of treatment weighting (IPTW). IPTW was constructed using the average treatment effect (ATE) framework. Red circles indicate covariate balance before weighting, and green circles indicate covariate balance after IPTW adjustment. The vertical dashed line at an absolute SMD of 0.10 denotes the prespecified threshold for acceptable balance.


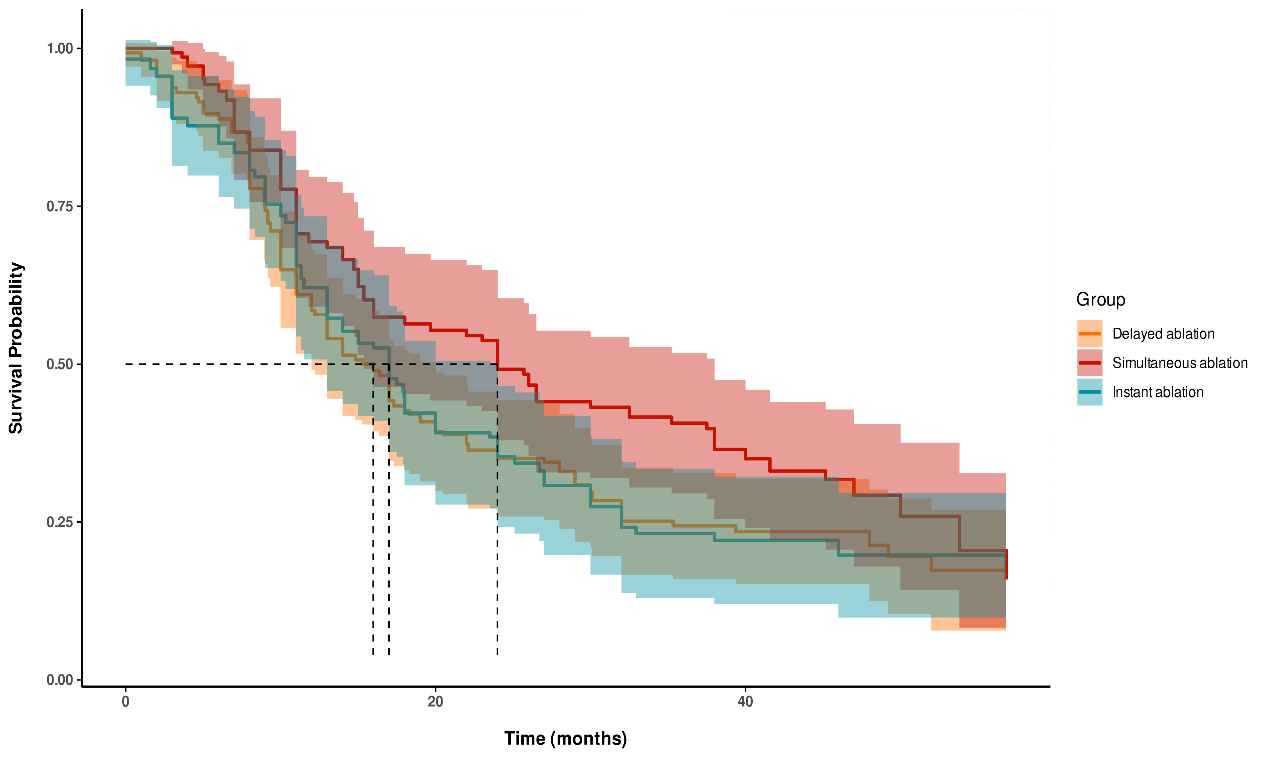


**Figure S4** **IPTW-weighted Kaplan-Meier curves for progression-free survival according to the timing of ablation relative to systemic therapy**

**
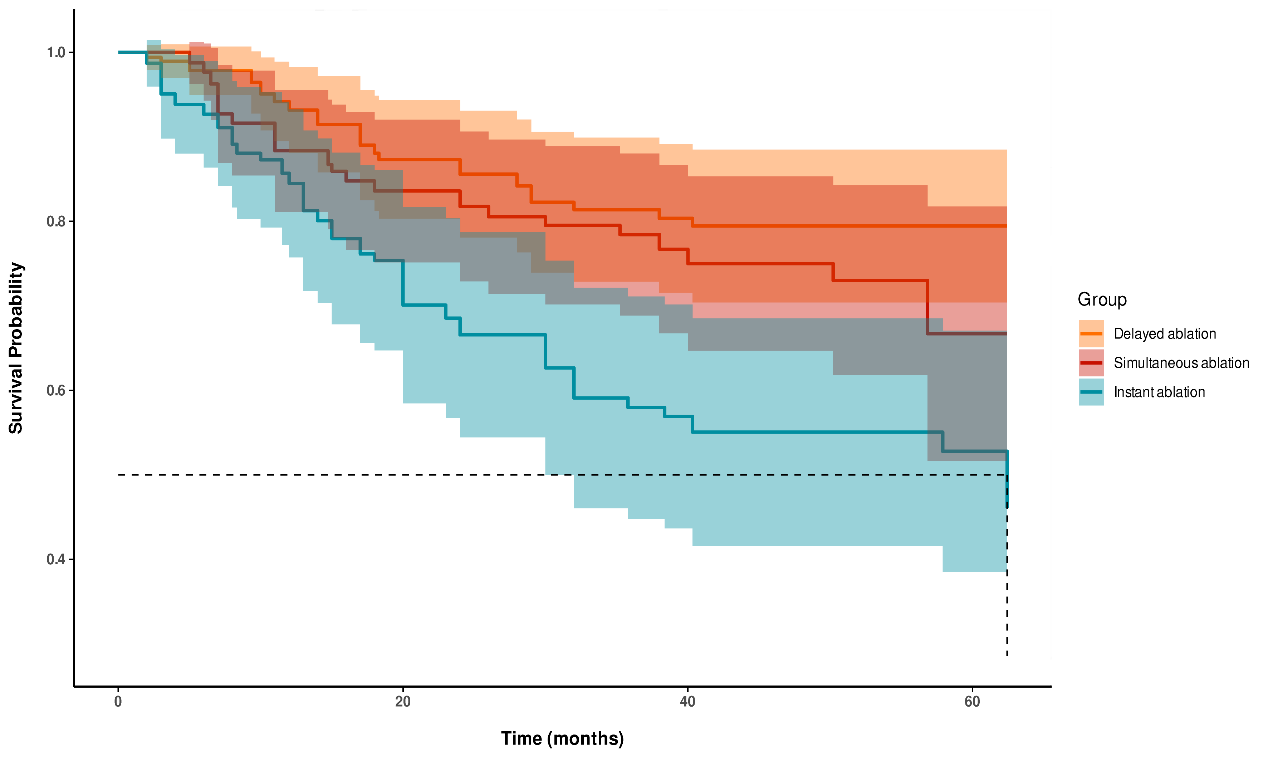
**

**Figure S5** **IPTW-weighted Kaplan-Meier curves for local tumor progression-free survival according to the timing of ablation relative to systemic therapy**


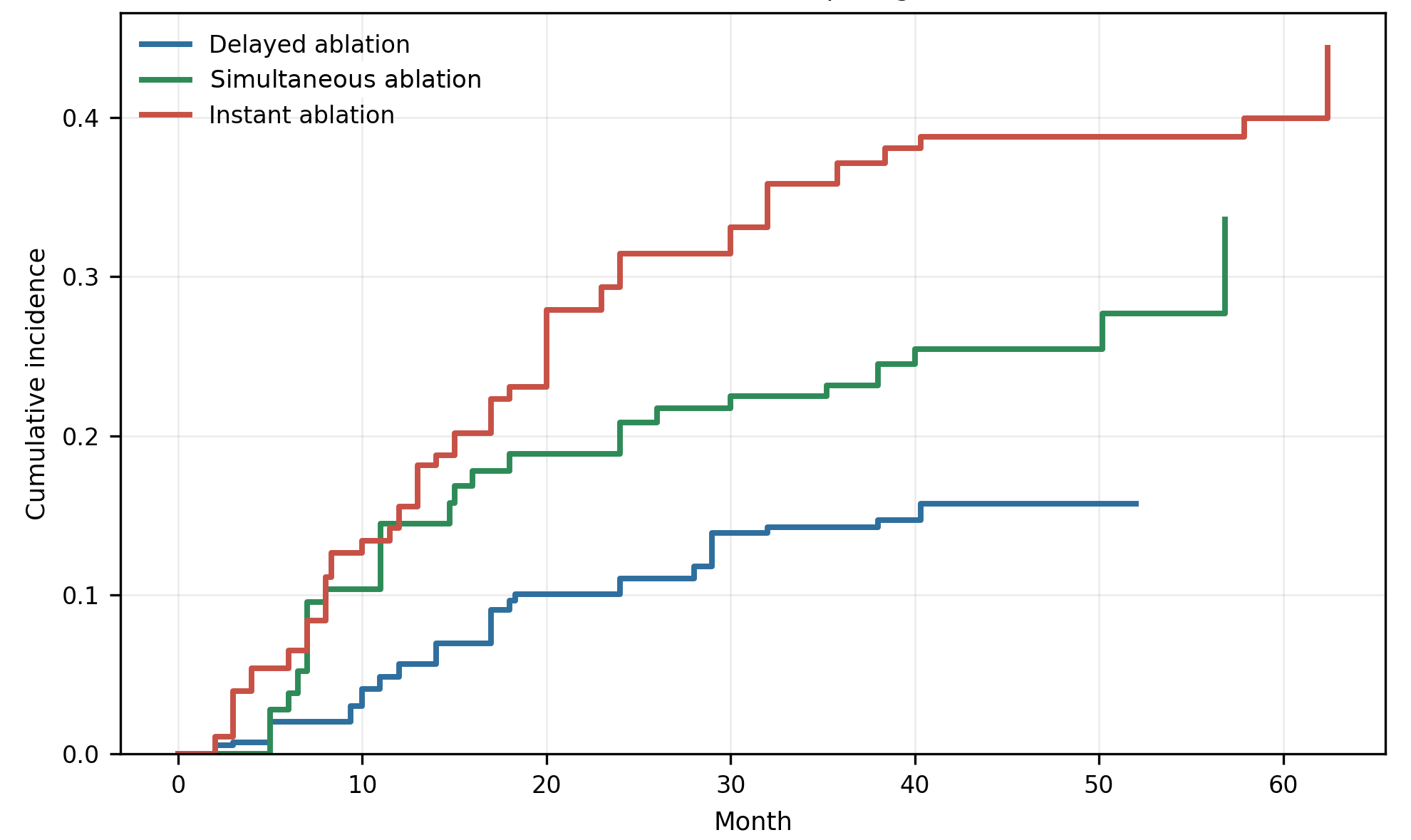


**Figure S6. IPTW-weighted cumulative incidence curves for local tumor progression-free survival with death treated as a competing event.** Death before local tumor progression-free survival was treated as a competing event. Cumulative incidence functions were estimated for local tumor progression according to treatment sequence after IPTW adjustment.
